# Effects of multiple doses of montmorillonite, alone and in combination with activated charcoal, on the toxicokinetics of a single dose of digoxin in rats

**DOI:** 10.22038/IJBMS.2023.66843.14661

**Published:** 2023

**Authors:** Fatemeh Heydarian, Mohammad Moshiri, Ali Roohbakhsh, Maryam Akaberi, Atoosa Haghighizadeh, Ameneh Ghadiri, Negar Yeganeh Khorasani, Leila Etemad

**Affiliations:** 1 Faculty of Pharmacy, Mashhad University of Medical Sciences, Mashhad, Iran; 2 Department of Clinical Toxicology, Imam Reza Hospital, Mashhad University of Medical Sciences, Mashhad, Iran; 3 Pharmaceutical Research Center, Pharmaceutical Technology Institute, Mashhad University of Medical Sciences, Mashhad, Iran; 4 Department of Pharmacodynamics and Toxicology, School of Pharmacy, Mashhad University of Medical Sciences, Mashhad, Iran; 5 Department of Pharmacognosy, School of Pharmacy, Mashhad University of Medical Sciences, Mashhad, Iran; 6 Department of Pharmaceutical and Food Control, School of Pharmacy, Mashhad University of Medical Sciences, Mashhad, Iran; 7 Student Research Committee, Faculty of Pharmacy, Mashhad University of Medical Sciences, Mashhad, Iran; 8 Medical Toxicology Research Center, Mashhad University of Medical Sciences, Mashhad, Iran; 9 International UNESCO Center for Health-Related Basic Sciences and Human Nutrition, Faculty of Medicine, Mashhad University of Medical Sciences, Mashhad, Iran

**Keywords:** Antidotes, Bentonite, Charcoal, Digoxin, Poisoning, Toxicokinetics

## Abstract

**Objective(s):**

A narrow margin between the therapeutic and toxic doses of digoxin can result in an increased incidence of toxicity. Since digoxin has an enterohepatic cycle, multiple oral doses of absorbents like montmorillonite may be useful in the treatment of digoxin toxicity.

**Materials and Methods:**

In this study, 4 groups of 6 rats received intraperitoneal digoxin (1 mg/kg), and half an hour later, distilled water (DW) or oral adsorbents, including montmorillonite (1 g/kg), activated charcoal (1 g/kg) (AC) alone or in combination in the ratio of 70:30. Half of the mentioned doses were also gavaged at 3 and 5.5 hr after digoxin injection. The serum level of digoxin, biochemical factors, and activity score were assessed during the experiment. Three control groups only received DW, montmorillonite, or AC.

**Results:**

All adsorbents were able to significantly decrease the serum level of digoxin compared to the digoxin+DW group (*P<*0.01). Only montmorillonite reversed the digoxin-induced hyperkalemia (*P<*0.05). Multiple dose administration of adsorbents also significantly reduced the digoxin area under the curve and half-life and increased digoxin clearance (*P<*0.05). However, there was no significant difference in the kinetic parameters between groups that received digoxin plus adsorbents.

**Conclusion:**

Multiple-dose of montmorillonite reversed digoxin toxicity and reduced serum digoxin levels by increasing the excretion and reducing the half-life. Montmorillonite has also corrected digoxin-induced hyperkalemia. Based on the findings, a multiple-dose regimen of oral montmorillonite could be a suitable candidate for reducing the toxicity issue associated with drugs like digoxin that undergo some degree of enterohepatic circulation.

## Introduction

Digoxin, in combination with other drugs, is indicated in the management of mild to moderate heart failure. It acts by increasing heart muscle strength and myocardial contractility. It is also used to treat certain types of supraventricular arrhythmias, such as chronic atrial fibrillation. Due to the narrow therapeutic index of digoxin (0.5-0.9 ng/ml), a slight change in its kinetic properties can cause large changes in the serum level and thus toxicity (1). Digoxin poisoning is very common in children and adults due to suicidal or accidental use of the pill, miscalculation of the dose, or drug-drug interactions. Acute and chronic digoxin poisoning can lead to several health problems, including gastrointestinal (nausea, vomiting, and diarrhea), and neurological (lethargy and confusion) complications. Cardiac disturbances are the most concerning and can be fatal (2). 

Digoxin-specific fab antibody fragments (Digifab) are the most effective medication used in the management of digoxin toxicity. However, Digifab is an expensive drug, it may not be readily available in some countries and therefore should be reserved for those patients with severe toxicity. One of the most common agents for the treatment of digoxin toxicity is activated charcoal (AC), which is especially useful for patients who have taken digoxin in less than 6 hr and when digifab is not available (3). Since digoxin has an enterohepatic cycle, multiple oral doses of AC may be useful to reduce the drug serum concentration (4).

The members of the smectite group, especially montmorillonite (MM), absorb various materials through the surface area or inner-plates distance of clay minerals. This property of clays gives them a high ability to absorb organic and inorganic molecules which makes them suitable for pharmaceutical applications (5, 6). Clay minerals are probably useful in the removal of toxins in the body. It was documented that MM can reversibly bind to digoxin *in vitro *(7). Smectite group minerals, especially MM, with absorbing properties of organic and inorganic substances, can be considered a good candidate for the treatment of toxicity. This study aims to evaluate the effect of multiple doses of MM or AC alone or in combination on the toxicokinetics of a single dose of digoxin in rats 

## Materials and Methods

***Animals and drugs***

Adult male *Wistar* rats weighing 240±10g were purchased from the Laboratory Animal Unit of Mashhad University of Medical Sciences (MUMS). Animals were kept at a controlled temperature (22±3 ^°^C), 12:12 hr light cycle, and allowed free access to food and water. 

The experiments were approved by the Animal Care Committee of Mashhad University of Medical Sciences, Mashhad, Iran, with approval ID: IR.MUMS.PHARMACY.REC.1398.019. 

***Experimental design***

**Figure 1:**
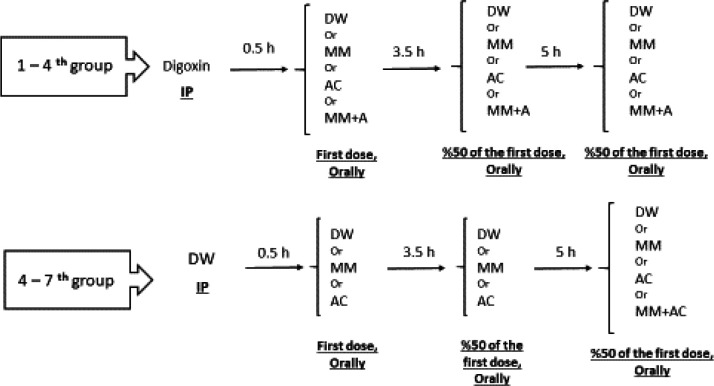
Schematic diagram of group design. The rats were divide into seven groups

IP: intraperitoneal; DW: distilled water; MM: montmorillonite; AC: activated

Forty-two rats were divided into seven groups and each group included six animals. Four groups received 1 mg/kg digoxin injectable solution (Rayan Darou, Iran) by IP injection, and half an hour later distilled water (DW) or adsorbents including MM (1 g/kg)( Sigma, Germany), AC (1 g/kg) (Merck, Germany), or MM+AC in the ratio of 70:30 (1 g/kg), through oral administration. They also received 50% of mentioned doses of DW or absorbents at 3 and 5.5 hr after digoxin treatment through the same route. The other three groups only received DW (distilled water) (Control), MM, or AC (Figure 1). 

***Serum digoxin level (SDL) and biochemical parameters evaluation***

For evaluation of digoxin, sodium, calcium, phosphorus, creatinine, and urea serum levels, the blood samples were obtained from the retro-orbital sinus of the animal at 1, 2, and 4 hr and through heart puncture at 6 hr after digoxin injection. Potassium serum level was also evaluated only at the 6^th^ hour. Biochemical parameters were measured by a standard autoanalyzer in the laboratory of Imam Reza Hospital, Mashhad, Iran.

***Activity evaluation***

The activity score of animals was recorded based on the following scale: 1: no movement, 2: stretching movements in response to painful stimulation, 3: stretching movement in response to stimulation, and 4: normal movement (6).

***Evaluation of the area under the curve, half-life, and clearance***

PK (pharmacokinetic) Solver 2.0 software, which works under Excel, was used for pharmacokinetic evaluations, and the trapezoidal method was used to evaluate the area under the curve (AUC). The clearance of digoxin was calculated as Clearance=(Dose*F)/AUC, where AUC is the area under the curve, Dose is the dose administrated to rats (1 mg/kg), and F is the bioavailability of the drug, which was considered equal to 100% (8).

The half-life is denoted by (t_1/2_) and is obtained from the following formula: where CL=clearance and Vd=volume of distribution.

$t\frac{1}{2}=0.7*\frac{\mathrm{Vd}}{\mathrm{Cl}}$

***Statistical analysis***

Kinetic calculations for each rat were done based on the reported concentrations, and then the mean of the relevant kinetic factors as well as biochemical factors was compared between groups. The results were reported as Mean±SEM. The means of biochemical parameters at different times were compared by one-way repeated measure ANOVA. The statistical analysis of mean values of potassium and calculated pharmacokinetic parameters were done by one-way ANOVA followed by the Tukey-Kramer test using the SPSS (ver. 16.0) software. For non-discontinuous quantitative variables (level of consciousness), non-parametric Kruskal-Wallis tests with Dunn’s test post were used. *P*-values ​​less than 0.05 were considered significant differences.

## Results

***Evaluation of serum digoxin levels***

**Figure 2:**
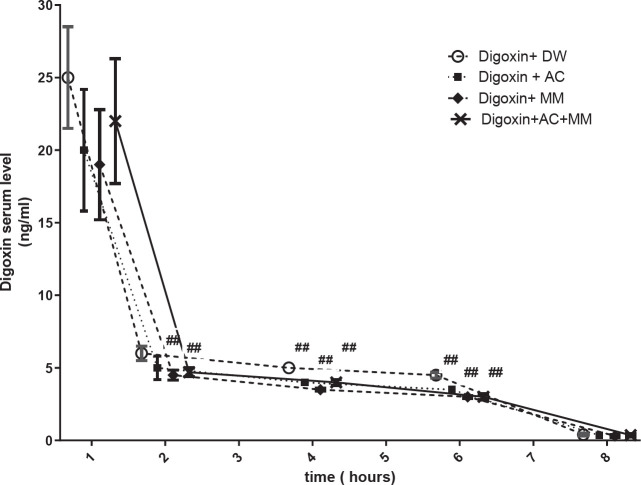
Comparison of serum digoxin level of digoxin-intoxicated rats, which were treated with digoxin (IP, 1 mg/kg) and different absorbents including distilled water (DW), MM (1 g/kg), AC (1 g/kg), or MM+AC (70:30, 1 g/kg)

SDL was evaluated at 1, 2, 4, and 6 hr after digoxin treatment. SDL increased at the first hour (25±3.5 ng/ml) and was similar in all treated groups (Figure 1). Oral administration of MM alone or in combination with AC decreased SDL at the second hour (*P*<0.01), while AC alone could not significantly reduce SDL compared with the digoxin group. However, all three adsorbents significantly reduced SDL at 4 and 6 hr after digoxin injection (*P*<0.01) (Figure 2).

***Blood*** ***biochemical*** ***assays***

One-way repeated measure ANOVA showed that the administration of digoxin did not induce any significant changes in blood levels of biochemical parameters including blood urea nitrogen (BUN), sodium, calcium, creatinine, and phosphorus in comparison with the control group and in different evaluating times (Table 1).

Regarding the effect of digoxin on blood potassium, digoxin injection was able to significantly increase the potassium level at the 6^th^ hour compared to the control group (*P*<0.01). Administration of MM alone or in combination with AC reduced the potassium serum level in comparison to the digoxin group (*P*<0.05). However, AC therapy could not decrease the serum level of potassium. Administration of AC, as well as MM alone, could not lead to a significant change in the serum biochemical parameter levels.

***Effect of adsorbents on the area under the curve, half-life, and clearance of digoxin***

Multiple-dose administration of adsorbents significantly reduced the area under the curve (AUC) of SDL (*P*<0.05). However, there was no significant difference between the AUC of groups that received DIG plus MM, AC, or MM+AC (Table 2).

The half-life of digoxin after administration of MM, AC, or MM+AC (70/30) was significantly reduced by 25.2% (*P*<0.05), 29.1% (*P*<0.05), and 38.7% (*P*<0.01), respectively. No significant differences in digoxin half-live were observed between the groups that received adsorbents.

The mean of digoxin clearance had also statistically increased in rats treated with AC, MM, and MM+AC (70/30) in comparison with the digoxin group (*P*<0.05) (Table 2). Multiple doses of AC increased the clearance of the drug by an average of 38.4%, and MM alone or in combination with AC increased the clearance of the drug by 76%. Statistical analysis did not show significant differences in digoxin clearance between the various absorbents treated groups (Table 2).

A full dose of the absorbents was gavaged 0.5 hr, followed by a half dose 3 and 5.5 hr after digoxin injection. Data are expressed as Mean±SEM; **P<0.01 and *P<0.05 compared to the DIG+DW group. # ratio to DIG+DW group, AUC: area under the curve, DIG: digoxin, AC: activated charcoal, and MM: montmorillonite

***Activity score***

No significant differences were observed between groups in terms of activity scores. Digoxin administration could not change the mean activity score and all animals received a score of four and were completely normal.

## Discussion

Digoxin is one of the cardiac glycosides that is prescribed for patients with heart failure and certain arrhythmias. In addition to numerous complications, including gastrointestinal, cardiovascular, and neurological problems, digoxin toxicity is very common due to its narrow therapeutic index (9). Multiple-dose administration of absorbents is used to treat poisoning when the drug or toxin has characteristics such as low volume of distribution, low protein binding capacity, and long clearance. Digoxin has a volume of distribution of about 5–7 liters/kg, 25% protein binding, and an elimination half-life of about one and a half days (36 hr) (10). Since digoxin has two of the above criteria and undergoes enterohepatic circulation, administration of multiple doses of AC, as well as other sorbents such as MM, are recommended in situations where Fab-DIG antibodies are not available or the patient has renal insufficiency (3, 11). 

Multiple-dose AC has long been known as a factor in increasing the excretion of absorbed toxins and drug compounds (11-13). This kind of treatment is known as “infinite sink” or “intestinal dialysis” (14, 15). In fact, in this method, an adsorbent substance is placed in the intestine and blood entering the intestines acts like a dialysis system. (12). In our previous studies, we found that MM could be an efficient and suitable alternative for absorbing substances with or without a high affinity to AC. MM is a type of clay that due to its adsorbent properties and cation exchange capacity can be used to treat poisoning with heavy metals and drugs, and some other toxins (5, 6, 15). 

The results of the present study showed that administration of MM, not AC, after 2 hr can significantly reduce the serum level of digoxin. However, MM, AC, and the combination of them significantly decreased SDL 4 and 6 hr after digoxin administration. Multiple-dose MM or AC, alone or in combination, have also been able to increase the clearance and decrease the half-life of digoxin. One study found that the use of AC could reduce serum digoxin levels by 30 to 40 percent in 12 to 18 hr (16). In a study performed on patients, it was found that repeated administration of 225 g AC over 40 hr could affect the kinetics of intravenous digoxin. Kinetic analysis showed that AC increased digoxin clearance by 47% and also reduced its half-life (17). In another study, the effect of MM alone and MM/surfactant hybrid nanocomposites containing MM 135-55 mg/kg and polysorbate 20 on the absorption and kinetic parameters of digoxin after 2, 3, 4, and 6 hr were investigated. The results showed that oral administration of MM and not the hybrid form, in addition to reducing the serum level of digoxin, led to reducing changes in the pharmacokinetic parameters including AUC0-6h, Cmax, C15 min, and half-life of digoxin (18). It was documented that MM, silica, and alumina surfaces are deprotonated as they enter the duodenum and small intestine and as the pH of the gastrointestinal tract increases, allowing more digoxin to bind (19). The effect of pH on the uptake of digoxin by MM has also been demonstrated in previous studies. X-ray diffraction studies have shown that digoxin was adsorbed onto MM by a reversible adsorption mechanism at pH 2 and 6. Digoxin binds to MM at pH 6 (similar to intestinal pH) with higher affinity than pH 2 (7). 

The results of the present study confirm the findings of previous studies on the role of MM in improving the toxicity of various toxic substances such as heavy metals, cyanide, and drug poisoning that were performed *in vitro* and in vivo. For example, oral administration of MM has been shown to reduce serum lead concentrations in the pig model (20, 21) and to reduce excess serum iron concentrations in rats (6).

Acute toxicity of digoxin requires oral administration of AC at doses of 25 to 100 g, which can cause constipation as a side effect (22). Although we did not use any visual grading system to assess the stool appearance, in line with our previous study, no changes were seen in stool consistency and frequency in the MM and AC-treated groups (5, 6). 

The specific anti-digoxin antibody is a well-known medication used in the management of digoxin toxicity (22, 23). However, anti-digoxin polyclonal antibodies are expensive and not easily available in all regions. As the elimination half-life of the digoxin-Fab complex is 24-72 hr, a long time is also needed to completely remove digoxin from the blood (24). In comparison, using MM or AC adsorbents can be a cost-effective method that does not meet these problems.

In the present study, no significant differences were observed in biochemical parameters of urea, sodium, calcium, phosphorus, and creatinine between animals receiving adsorbents plus digoxin or digoxin alone. However, the level of potassium was increased significantly in the digoxin group. Hyperkalemia is common in acute and chronic digoxin toxicity that can exacerbate the toxic effects of digoxin on the myocyte and increase the risk of heart arrhythmias (25). On the other hand, a decrease in the potassium level was indicated as a side effect of clay ingestion, due to the high affinity of clay minerals and thus decreased GI absorption of potassium (16, 19, 26). In this study, administration of MM alone or in combination with AC prevented digoxin-induced hyperkalemia. Hypokalemia was not observed in the group that received only MM. Therefore, the effect of MM on digoxin-induced hyperkalemia may be caused by two mechanisms: 1) indirect effect of MM on reducing digoxin absorption and 2) direct effect of MM on potassium absorption in the intestines (27). Digoxin is mostly excreted by the kidneys and to a lesser extent through hepatic routes (16). It is noticeable that increases in digoxin serum levels can be influenced by several factors, including electrolyte abnormalities and changes in renal function (renal clearance, serum creatinine, and urea levels). However, in this study, digoxin at the selected dose did not affect serum urea and creatinine levels and thus did not affect kidney function.

**Table 1:** Levels of biochemical parameters in digoxin-intoxicated rats that were treated with digoxin (IP, 1 mg/kg) and different compounds including distilled water (DW), MM (1 g/kg), AC (1 g/kg), or MM and AC in combination ratio of 70:30

Biochemical factors	Time (hrs)	Control	DIG+DW	DIG+AC	DIG + MM	DIG+MM +AC	MM	AC
BUN (mg/dl)	1	75 ± 2.5	77 ± 3	73 ± 2	70 ± 2.3	72 ± 4	65 ± 5	79 ± 3.5
	2	76 ± 2.3	74 ± 3.3	70 ± 2.1	68 ± 2.4	73 ± 3.5	72 ± 4.9	76 ± 4.6
	4	73 ± 3.2	79 ± 3.6	75 ± 3.3	74 ± 3.4	77 ± 3.8	70 ± 3.4	70 ± 6.4
	6	77 ± 2.6	81 ± 4.7	79 ± 3.9	73 ± 4.5	70 ± 4.7	74 ± 3.4	72 ± 3.4
Sodium (mEq/l)	1	138 ± 8.7	142 ± 9.4	146 ± 8.5	141 ± 9.6	139 ± 7.6	141 ± 9.1	140 ± 7.5
	2	140 ± 8.4	141 ± 9	145 ± 9.2	140 ± 9.2	138 ± 8.5	142 ± 8.7	142 ± 8.4
	4	141 ± 8.5	143 ± 8.6	144 ± 9.1	139 ± 8.6	140 ± 8.8	139 ± 7.6	138 ± 8.9
	6	139 ± 9.1	139 ± 8.5	139 ± 9.4	143 ± 9.4	141 ± 7.5	138 ± 8.5	143 ± 9.1
Calcium (mg/dl)	1	9.1 ± 0.4	9.3 ± 0.6	8.7 ± 0.7	8.5 ± 0.8	9.5 ± 0.9	9.3 ± 0.8	9.2 ± 0.7
	2	9.3 ± 0.3	9.4± 0.5	8.9 ± 0.3	8.8 ± 0.3	9.4 ± 0.8	9.1 ± 0.4	9.5 ± 0.9
	4	8.9 ± 0.5	9.2 ± 0.6	9.0± 0.5	9.0 ± 0.4	9.1 ± 0.5	9.0 ± 0.6	9.4 ± 0.4
	6	9.0 ± 0.4	9.3 ± 0.7	8.6 ± 0.8	8.9 ± 0.5	9.3 ± 0.7	9.4 ± 0.9	9.2 ± 0.8
Creatinine (mg/dl)	1	0.7 ± 0.1	0.9 ± 0.2	1.2 ± 0.2	1.1± 0.3	1.0 ± 0.2	0.8 ± 0.3	1.2± 0.3
	2	0.8 ± 0.3	1.1 ± 0.4	0.9 ± 0.3	1.0 ± 0.3	1.1 ± 0.3	0.7 ± 0.2	1.2 ± 0.2
	4	0.9 ± 0.2	1.0 ± 0.3	1.0± 0.2	0.9 ± 0.2	0.9± 0.1	1.0 ± 0.1	1.1 ± 0.1
	6	1.1 ± 0.4	0.8 ± 0.3	1.1 ± 0.4	1.1± 0.4	0.9 ± 0.3	0.9 ± 0.3	1.0 ± 0.2
phosphorus (mg/dl)	1	8.5± 0.4	9.4 ± 0.7	8.4 ± 0.5	9.2± 0.6	8.6 ± 0.7	7.9 ± 0.5	7.8 ± 0.3
	2	9.1± 0.3	9.2± 0.6	8.9± 0.7	9.4± 0.5	8.9 ± 0.5	8.4 ± 0.6	8.3± 0.5
	4	8.8± 0.3	8.9 ± 0.4	8.7± 0.4	9.3 ± 0.4	8.8 ± 0.6	8.7± 0.7	8.5 ± 0.6
	6	9.2 ± 0.4	9.4± 0.5	9.0 ± 0.6	9.4 ± 0.4	8.9± 0.4	8.9 ± 0.4	8.3 ± 0.7
Potassium (mg/dl)	6	5.2 ± 0.2	6.9± 0.3**	6.6 ± 0.34	5.4± 0.23#	5.6 ± 0.43#	5.8 ± 0.3	5.7 ± 0.3

Absorbents were gavaged in full dose 0.5 hr and in half dose 3 and 5.5 hr after digoxin injection. The results are reported as mean±SEM, **P<0.01 compared to the control group and ^##^*P<*0.01 compared to the digoxin group

DW: distilled water; MM: montmorillonite; AC: activated; DIG:Digoxin

The absorbents were gavaged in full dose half an hour and in half doses 3 and 5.5 hr after digoxin injection. The results are reported as mean±SEM, ^##^*P<*0.01 compared to the DW group

DW; Distilled Water, AC; Activated Charcoal, MM; Montmorillonite

**Table 2:** Pharmacokinetic parameters of digoxin in rats that were treated with different compounds including distilled water (DW), MM (1 g/kg), AC (1 g/kg), or MM and AC in combination ratio of 70:30

Group	t 1/2 (hour)		AUC (ng/ml*h)			Clearance (mg/kg)/(ng/ml)/h)		
	**Mean±SEM**	**(%)#**	**Mean±SEM**	**(%)#**	**Mean±SEM**		**(** **%)#**
DIG+DW	9.63± 0.7	100	111.0691± 9.7	100	0.009003± 0.0004		100
DIG+AC	7.2 ± 0.65*	74.8	80.25143 ± 7.6*	72.3	0.012461± 0.0009*		138.4
DIG+MM	6.83± 0.54*	70.9	68.34564 ± 6.3**	61.5	0.014632± 0.0018**		162.5
DIG+MM+AC	5.9 ± 0.5**	61.3	62.99138± 5.9**	56.7	0.015875± 0.0011**		176.3

A full dose of the absorbents was gavaged 0.5 hr, followed by a half dose 3 and 5.5 hr after digoxin injection. Data are expressed as Mean±SEM; **P<0.01 and *P<0.05 compared to the DIG+DW group. # ratio to DIG+DW group

AUC: area under the curve, DIG: digoxin, AC: activated charcoal, and MM: montmorillonite

## Conclusion

Taken together, the present work provides us with an idea about the multiple-dose administration of MM alone or in combination with AC for the treatment of digoxin intoxication. The results showed that multiple-dose adsorbents reduced serum digoxin levels by increasing its excretion and reducing its half-life. However, only MM could reverse the digoxin-induced hyperkalemia. Therefore, multiple-dose MM is a good option to reduce the side effects of digoxin overdose or poisoning. However, it needs to be confirmed by further research. 

## Authors’ Contributions

L E, M M, and A R conceived the original idea. F H and A H performed experiments. L E, M M, and A R supervised the research. M A, A H, and A G analyzed the data. F H, N YK, M A, A H, and A G wrote the original draft. L E and M M edited final draft.

## Conflicts of Interest

The authors declare that there are no conflicts of interest.
